# Development of Anti-Icing and Skid-Resistant Road Surfaces Using Methyl Methacrylate (MMA) Resin-Based Composites

**DOI:** 10.3390/ma18030501

**Published:** 2025-01-22

**Authors:** Sung-Hyun Eom, Hyo-Seong Jeon, Tae-Gyue Ryue, Hun-Jae Lee, Hong-Gi Kim, Tadesse Natoli Abebe

**Affiliations:** 1 Dream Indesign Co., Ltd. 1802, 3 Gongwon-ro, Guro-gu, Seoul 08378, Republic of Korea; esh905e@naver.com; 2 Korea Testing & Research Institute, 8, Techno saneop-ro 29beon-gil, Nam-gu, Ulsan 44776, Republic of Korea; indesign24@naver.com (H.-S.J.); rtg@ktr.or.kr (T.-G.R.); imfin@ktr.or.kr (H.-J.L.); 3 Civil and Environmental Engineering Department, Hanyang University, Jaesung Civil Engineering Building, 222 Wangsimni-ro, Seongdong-gu, Seoul 04763, Republic of Korea

**Keywords:** MMA, anti-icing, substitution, resin, additive material

## Abstract

Winter road safety is significantly compromised by ice formation, leading to increased vehicular accidents due to reduced friction. Traditional anti-icing strategies, such as chemical deicers, present environmental and structural drawbacks, necessitating innovative solutions. This study evaluates methyl methacrylate (MMA)-based resin composites for anti-icing and skid-resistant applications. These composites are particularly intended for application on asphalt and concrete pavements in urban roads, highways, and other high-traffic areas prone to icing during winter. MMA composites exhibit excellent mechanical properties, including tensile strength of up to 10 MPa and compressive strength of 34 MPa under optimized formulations. These composites are specifically developed for application on asphalt and concrete pavements commonly found in urban roads, highways, and other high-traffic areas, where icing and skid resistance are critical challenges during winter conditions. Anti-icing performance was enhanced by incorporating additives like magnesium chloride hexahydrate, achieving a freezing point reduction to −12.9 °C and a heat of solution of 0.429 kJ/g. Laboratory tests revealed that increasing anti-icing additives reduced ice adhesion and melting time, with a trade-off in compressive strength, which decreased from 30 MPa (unmodified) to 16 MPa at higher additive concentrations. Skid resistance was improved through the addition of high-friction aggregates, ensuring durability under icy and wet conditions. These results highlight MMA composites as a sustainable and cost-effective alternative to traditional deicing methods, offering enhanced road safety and reduced environmental impact. Further research is recommended to optimize formulations and validate performance through field trials under varying climatic conditions.

## 1. Introduction

The deterioration of road safety during winter is a significant challenge due to ice formation and reduced skid resistance caused by snow and frozen precipitation. Road surfaces covered with ice significantly increase the risk of vehicular accidents due to reduced friction, leading to unsafe driving conditions [1]. Anti-icing strategies have been implemented globally to mitigate this issue, primarily through the application of chemical deicers, such as sodium chloride, calcium chloride, and magnesium chloride [2]. However, these traditional methods are associated with several drawbacks, including environmental degradation, corrosion of vehicles and infrastructure, and frequent reapplication requirements during prolonged snowfall [3,4]. Consequently, the development of innovative, sustainable, and long-lasting anti-icing technologies has become a subject of critical interest in transportation engineering.

In this context, polymer-based materials have gained attention as an alternative for enhancing road surface performance under adverse weather conditions [5]. Among these, methyl methacrylate (MMA)-based composites have emerged as promising candidates due to their excellent mechanical strength, high durability, chemical resistance, and strong bonding with asphalt and concrete substrates [6,7]. MMA composites exhibit superior resistance to wear and environmental factors compared to traditional binders. The proposed MMA resin-based composites are engineered for compatibility with a variety of pavement types, including asphalt, concrete, and mastic asphalt. These surfaces represent the primary road materials used in urban and rural infrastructure, allowing the composites to be employed across diverse climatic and structural conditions, making them particularly suitable for harsh climatic conditions and heavy-traffic zones [8]. Furthermore, their rapid curing characteristics allow for minimal disruption during construction and maintenance activities, which is a critical requirement in transportation infrastructure projects [9]. Anti-icing performance can be achieved by modifying road surfaces to prevent ice adhesion and reduce the accumulation of frozen precipitation. Ice-phobic surfaces, which exhibit low adhesion to ice, have been extensively investigated in the context of aerospace and energy industries [10]. By incorporating functional materials, such as hydrophobic polymers, nanoparticles, and micro-textured patterns, the adhesion of ice to a surface can be minimized [11]. This principle has been adapted to road pavements by integrating anti-icing additives, which disrupt ice formation or facilitate its removal under vehicular loads [12]. In this study, MMA resin-based composites were enhanced with additives such as butyl acrylate, 2-hydroxyethyl methacrylate (HEMA), and magnesium chloride hexahydrate. These modifications reduced the freezing point to −12.9 °C and improved ice-melting performance without significantly compromising the mechanical properties of the material.

Another critical consideration for winter road safety is skid resistance, which directly influences the frictional performance of the pavement surface. Skid resistance is primarily determined by the macrotexture and microtexture of the pavement. Over time, road surfaces undergo wear, polishing, and the loss of aggregate exposure, leading to a decline in frictional properties. The addition of high-friction aggregates or textured coatings to road surfaces is a common strategy to improve skid resistance [13]. MMA resins, when combined with aggregates such as bauxite, silica, or other high-hardness particles, can enhance the macrotexture of the road surface, thereby improving skid resistance under wet and icy conditions [14]. Additionally, the flexibility of MMA composites allows for the incorporation of surface treatments that optimize both anti-icing and skid-resistant functionalities [15]. Previous studies have explored various methods for improving road surface anti-icing properties, including hydrophobic coatings, phase-change materials, and electrical heating systems [16,17]. However, these approaches often present limitations, such as short service life, high installation costs, and energy-intensive operations. Polymer-modified surfaces, particularly MMA-based systems, offer an attractive alternative due to their cost effectiveness, durability, and ease of application [17]. The use of MMA resin composites for anti-icing applications has also been supported by advancements in material science, such as the development of nanocomposites and functionalized additives that improve ice-repellency and mechanical strength [18].

Environmental and economic factors also play a significant role in the adoption of road surface technologies. The frequent use of chemical deicers contributes to groundwater contamination, soil salinization, and damage to vegetation [19]. These environmental impacts necessitate the exploration of sustainable materials that reduce or eliminate the need for repeated chemical treatments. MMA resin-based composites, when integrated with anti-icing and skid-resistant features, provide a long-term solution that minimizes environmental damage while maintaining road safety standards. Additionally, the durability and low maintenance requirements of MMA-based systems present significant economic benefits by reducing lifecycle costs and extending pavement service life [20]. The effectiveness of MMA resin composites in enhancing road performance is influenced by their formulation, surface texture, and the addition of functional fillers or aggregates. To optimize these systems for anti-icing and skid resistance, comprehensive investigations are required to analyze the interplay between material composition, surface characteristics, and environmental performance. Recent developments in nanotechnology, such as the incorporation of silica nanoparticles, graphene, or carbon-based materials, offer new opportunities to enhance the ice-phobic and frictional properties of MMA composites [21,22]. Despite significant progress, research on the application of MMA resin composites for anti-icing and skid-resistant road surfaces remains limited.

This study aims to address these research gaps by developing and evaluating MMA resin-based composites for anti-icing and skid-resistant road surfaces. Specifically, the objectives of this research include (1) the formulation of MMA resin composites with anti-icing additives and high-friction aggregates, (2) the characterization of their mechanical, thermal, and surface properties, and (3) the evaluation of their anti-icing and performance through laboratory tests and field trials. By integrating advanced material design with practical performance testing, this study seeks to provide a comprehensive solution to winter road safety challenges.

## 2. Experimental Procedure

### 2.1. Materials and Reagents

Methyl methacrylate (MMA, >99%), butyl acrylate (BA, >99%), and 2-hydroxyethyl methacrylate (HEMA, >99%) were obtained from commercial supplier (Sigma-Aldrich Inc. Gangnam-gu, Seoul, Republic of Korea). 2-Hydroxy-4-(octyloxy) phenyl methanone (>99%), paraffin wax (P-WAX, >99%), and 1-dodecyl mercaptan (NDM, >98%) were purchased from standard chemical suppliers (BLD Pharmatech Ltd., Shanghai 201601, China). Dimethyl-p-toluidine (>99%) was also used as received. For anti-icing studies, magnesium chloride hexahydrate (>98%), potassium acetate (>99%), potassium carbonate (>99%), and polyethylene glycol (PEG, Mw = 400) were obtained and used without further purification (Sigma-Aldrich Inc., Gangnam-gu, Seoul, Republic of Korea). These materials were selected based on their effectiveness in de-icing and anti-icing applications to study surface performance under sub-zero conditions.

Deionized water (conductivity: ≤0.10 μS cm^−1^) was prepared in the laboratory using a purification system to ensure consistent quality throughout the experiments. All chemicals and reagents were handled following standard safety protocols and were used as received without further treatment unless otherwise stated.

### 2.2. Preparation of MMA Based Resin and Mix Proportion

The MMA-based resin was developed through a stepwise process. First, methyl methacrylate (MMA) (Table 1, Content 1) was inserted into the mixing machine and heated to a temperature range of 70–80 °C. Once the MMA was heated, the subsequent components, including butyl acrylate, 2-hydroxyethyl methacrylate (HEMA), 2-hydroxy-4-(octyloxy) phenyl methanone, paraffin wax (P-WAX), and 1-dodecyl mercaptan (Table 1, Content 2–6), were added sequentially and thoroughly mixed to ensure homogeneity. After mixing, the resin was dried for 12 h to remove any moisture and enhance its stability. The dried mixture was then cooled to 50–60 °C, after which dimethyl-p-toluidine (Table 1, Content 7) was added and mixed thoroughly for each of the five prepared samples (Sample 1 to Sample 5 in Table 2).

The anti-icing mixtures were prepared based on the mixing ratios provided in Table 1, where different proportions of magnesium chloride hexahydrate, potassium acetate, potassium carbonate, and polyethylene glycol were combined. These mixtures were then evaluated to determine their heat of solution and freezing point, as shown in Table 3. The results indicated that Mix 7, derived from the ratios in Table 3, exhibited the best performance with a heat of solution of 0.429 kJ/g and a freezing point of −12.9 °C. This process ensured the development of a stable and effective MMA-based resin with optimal properties for anti-icing applications.

### 2.3. Experimental Methods and Methodology

#### 2.3.1. Mechanical Strength Test

To fully evaluate the MMA-based resin’s performance under a range of stress circumstances, a complete experimental investigation was carried out to measure its mechanical strength, including elongation at break, tensile strength, and bonding strength. Tests were carried out using the ASTM D6723 [23] standard to ascertain elongation at break. The percentage elongation, or the material’s capacity to deform before failing, was measured after specimens were prepared and put through a controlled tensile strain. This test is crucial for determining the resin’s ductility and flexibility. The ASTM D4867 [24] standard was followed while measuring the tensile strength. A uniaxial tensile load was applied to the specimens until they failed, and the highest stress the material could withstand was noted. This test reveals information about the resin’s resistance to tensile forces and highlights its structural integrity under tension. For bonding strength, the tests were performed based on the AASHTO T 361 [25] protocol. Specimens with bonded surfaces were prepared, and a shear load was applied to measure the maximum force required to cause debonding. The bonding strength results indicate the material’s adhesive performance and suitability for applications requiring strong interfacial bonding.

#### 2.3.2. Anti-Icing Test

**Figure 1:**
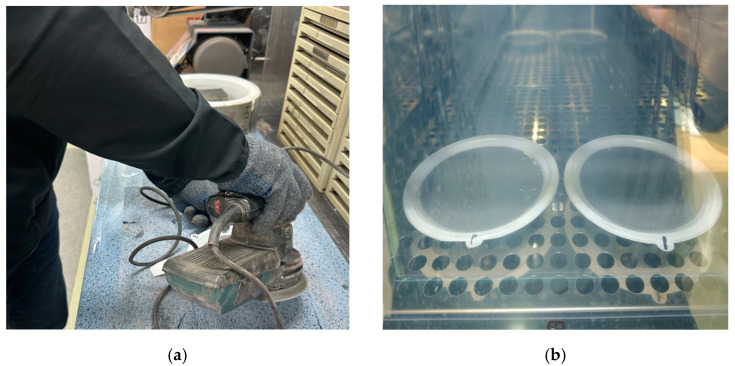
Anti-icing test: (**a**) surface treatment; (**b**) freezing sample in the chamber.

As can be seen in Figure 1a,b, the anti-icing test was conducted to evaluate the melting performance and compressive strength of the specimens under controlled conditions. First, the specimens underwent surface treatment to ensure uniformity and proper adhesion of water droplets during the test. A specific amount of water was then applied evenly to the treated surface of each specimen. The specimens were placed in a freezing chamber and maintained at −5 °C until the water completely froze. To ensure consistent ice formation, the frozen specimens were held in the chamber for 12 h. After the freezing process, the anti-icing test was performed at 0 °C, where the melting performance of eSach specimen was evaluated based on the melting time of the ice. Following the anti-icing test, the specimens were subjected to a compressive strength test to assess their mechanical properties after exposure to freezing and melting conditions.

## 3. Results and Discussion

### 3.1. Elongation at Break and Bonding Strength

**Figure 2:**
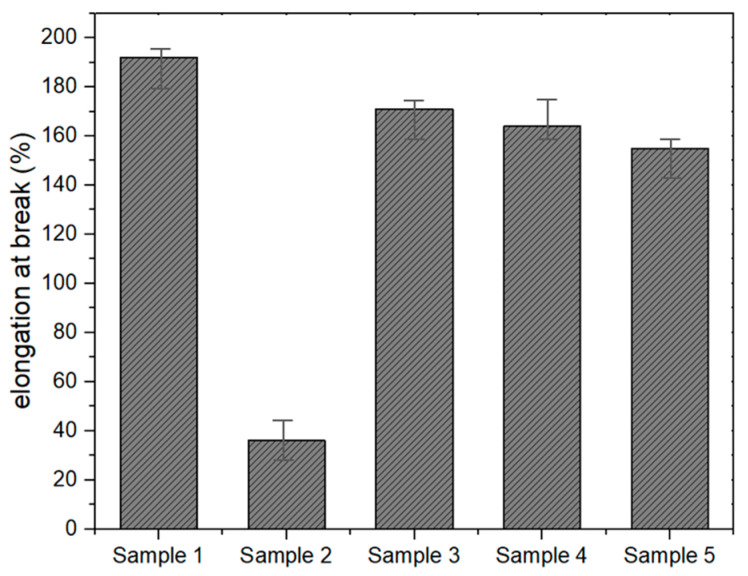
Test results of elongation at break.

Figure 2 shows the elongation at break (%) of MMA-based resin samples, which is influenced by the composition and presence of specific additives. Methyl methacrylate (MMA), the primary monomer, forms the backbone of the resin, providing structural stability, but it tends to be rigid on its own. The addition of butyl acrylate, known for its elasticity, introduces flexibility to the resin matrix, allowing for greater elongation under tensile stress [6]. Similarly, polyethylene glycol (PEG), a soft and flexible polymer, likely enhances ductility and reduces brittleness by acting as a plasticizer within the resin structure. The variation in elongation values across the samples can also be attributed to the influence of other components like paraffin wax (P-WAX) and 1-dodecyl mercaptan. Paraffin wax, with its lubricating properties, may improve the resin’s ability to deform under stress by reducing internal friction within the polymer chains [3,22]. Meanwhile, 1-dodecyl mercaptan, functioning as a chain transfer agent, controls the molecular weight of the polymer, which in turn impacts the mechanical properties, including flexibility and elongation.

Samples with higher elongation at break indicate a more flexible and well-distributed polymer network, likely facilitated by the synergistic effects of butyl acrylate and polyethylene glycol. On the other hand, lower elongation values suggest formulations where the resin composition lacked sufficient flexibility modifiers or had a less uniform molecular structure, leading to increased stiffness and reduced capacity to deform [26]. The consistent performance seen in some of the samples also suggests that fine-tuning the ratio of components plays a key role in optimizing elongation properties. The results highlight the critical role of component interactions, where structural monomers like MMA are complemented by additives such as butyl acrylate and PEG to achieve the desired mechanical flexibility. Additionally, paraffin wax and chain regulators like 1-dodecyl mercaptan influence the material’s overall elongation behavior, contributing to the observed variations in mechanical performance.

**Figure 3:**
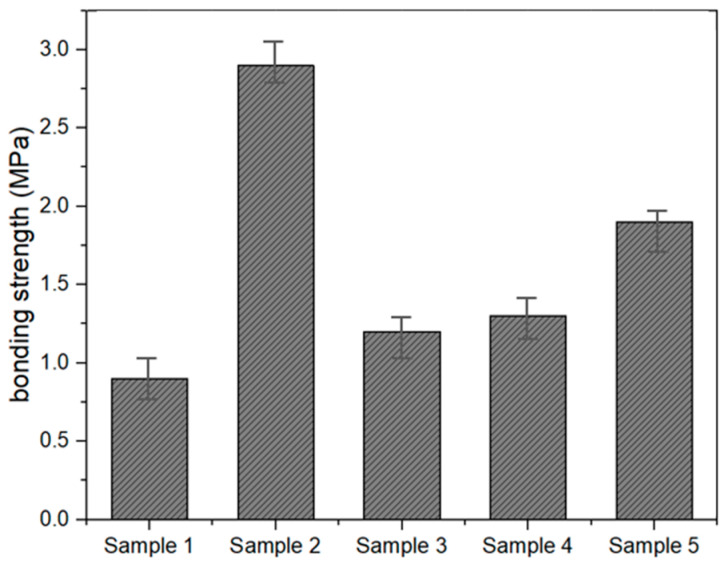
Test results of bonding strength.

Figure 3 illustrates the bonding strength (MPa) of MMA-based resin samples, showing clear differences in adhesive performance due to variations in formulation. Sample 2 displays the highest bonding strength, reaching 3.0 MPa, the high bonding strength observed indicates that the material can effectively adhere to road surfaces, minimizing the likelihood of cavitation or slippage under dynamic loads. The presence of 2-hydroxyethyl methacrylate (HEMA) likely contributes to this adhesion through improved intermolecular interactions. Further surface preparation measures, such as priming, can be considered in practical applications to enhance adhesion, which can be attributed to the presence of components like butyl acrylate and 2-hydroxyethyl methacrylate (HEMA). These additives improve flexibility and promote stronger intermolecular interactions, enhancing adhesion to the substrate [27]. The hydroxyl groups in HEMA, in particular, likely contribute to better bonding through hydrogen bonding. In contrast, Sample 1 exhibits the lowest bonding strength at approximately 1.0 MPa, indicating that its formulation lacks sufficient bonding-promoting components or flexibility modifiers, resulting in weaker adhesion. Samples 3, 4, and 5 show moderate bonding strength, ranging between 1.5 and 2.0 MPa. This suggests that their formulations achieve a balance between structural stability and adhesive properties but do not perform as well as Sample 2. The inclusion of paraffin wax (P-WAX) and 1-dodecyl mercaptan in these samples likely influenced the results by modifying the polymer network and molecular weight, factors that play key roles in determining bonding strength [12,28]. Overall, the bonding strength trends indicate that optimizing the mix ratios of structural monomers like MMA and flexibility-enhancing additives such as butyl acrylate and HEMA is essential for improving adhesion performance. Sample 2’s superior bonding strength highlights the importance of achieving a well-balanced composition that combines flexibility, molecular interaction, and structural integrity.

### 3.2. Mechanical Strength

**Figure 4:**
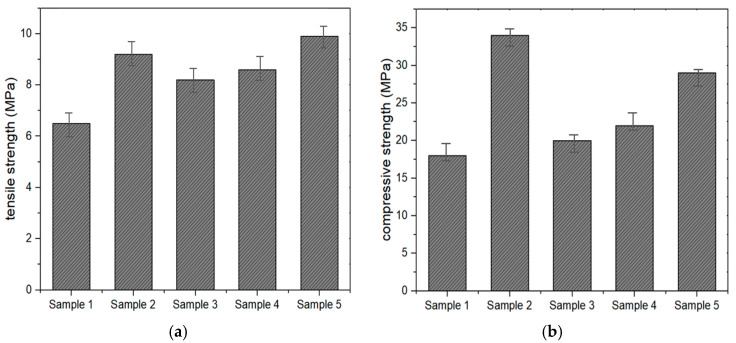
Mechanical properties: (**a**) tensile strength, and (**b**) compressive strength.

In Figure 4a, the tensile strength results show clear variations among the MMA-based resin samples. Sample 5 exhibited the highest tensile strength (10 MPa), indicating a well-formed polymer network with effective cohesion and load-bearing capacity. This can be attributed to the balanced interaction between methyl methacrylate (MMA) as the structural backbone and elasticity-enhancing additives like butyl acrylate. Sample 2, with tensile strength just below 9 MPa, also demonstrates a robust structure, likely due to efficient molecular distribution and chain entanglement [20]. In contrast, Sample 1 recorded the lowest tensile strength (6.5 MPa), suggesting a weaker network, potentially due to insufficient flexibility modifiers or reduced molecular weight caused by chain transfer effects from 1-dodecyl mercaptan. Samples 3 and 4, with intermediate values near 8 MPa, indicate a moderate balance between flexibility and strength, influenced by components like polyethylene glycol (PEG) and paraffin wax. These findings emphasize the need for precise control of additive ratios to optimize tensile properties.

The compressive strength, as shown in Figure 4b results, reveals significant differences in the samples’ resistance to compressive forces. Sample 2 achieved the highest compressive strength (34 MPa), likely due to enhanced molecular interactions and crosslinking, facilitated by components like 2-hydroxyethyl methacrylate (HEMA). HEMA’s hydroxyl groups improve stability under compression, contributing to this superior performance [29]. Sample 5, with a compressive strength of 30 MPa, maintains strong structural integrity, reflecting a good balance between rigidity and flexibility. In contrast, Sample 1 showed the lowest value (18 MPa), suggesting a less cohesive structure with lower resistance to compressive deformation. Samples 3 and 4, with mid-range values near 24 MPa, indicate moderate stability, influenced by additives such as paraffin wax, which can improve flexibility but may reduce compressive resistance at higher concentrations [30].

### 3.3. Anti-Icing Test Results

**Figure 5:**
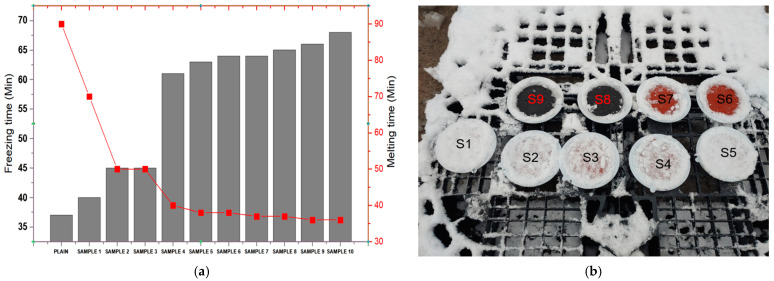
Anti-icing performance (**a**) performance results (**b**) melting efficiency results.

To evaluate the anti-icing performance, the selected anti-icing material (Mix 7) from Table 1. was incorporated into Sample 5 of the MMA-based resin. The anti-icing additive was added incrementally at ratios ranging from 0 g to 5 g in 0.5 g steps, and the results were analyzed to determine the impact on freezing time, melting time, and mechanical performance. As shown in the freezing and melting test results in Figure 5a,b, the addition of Mix 7 significantly influenced the freezing and melting behavior of the resin. Freezing time increased with the incorporation of the anti-icing material, demonstrating the material’s ability to slow down the ice formation process. At the same time, the melting time decreased considerably as more anti-icing additive was introduced, as evident from the graph. This suggests that the presence of components like magnesium chloride hexahydrate and potassium carbonate effectively disrupts the ice crystal structure, accelerating the melting process [26]. Additionally, the contribution of polyethylene glycol (PEG) enhances flexibility and thermal conductivity, further promoting faster melting while maintaining surface integrity.

The visual inspection of specimens in the second image clearly shows the effectiveness of the anti-icing additive used. Samples incorporating higher additive ratios exhibited less ice adhesion and faster melting times compared to untreated samples, indicating an improved anti-icing performance. Furthermore, the mechanical evaluation of the resin, specifically compressive strength, was performed after the anti-icing test [7,31]. Despite the addition of the anti-icing material, the compressive strength of the modified Sample 5 remained stable. Although specific freeze–thaw cycling tests were not conducted in this study, the stability of compressive strength after anti-icing treatment suggests that the proposed MMA resin-based composite is likely to maintain its structural integrity under freezing and melting conditions. This assumption is based on the material’s high bonding strength and resistance to ice-induced stresses, as demonstrated in the anti-icing tests, confirming that the structural integrity of the MMA-based resin was not significantly compromised. This stability highlights the compatibility of the anti-icing compound with the resin matrix, where the polymer network maintains its mechanical properties while enhancing anti-icing performance.

**Figure 6:**
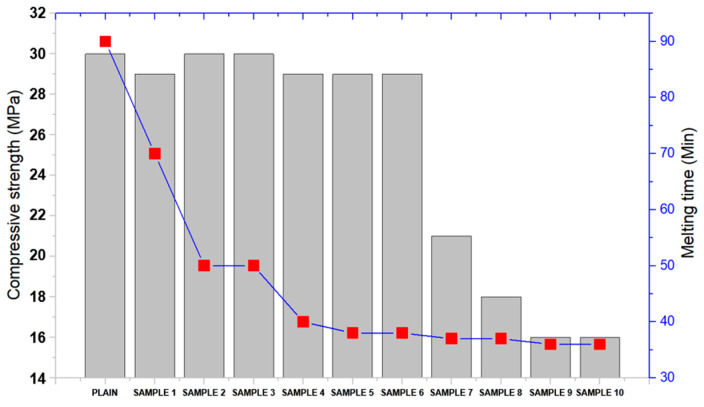
Compressive strength after melting.

The mechanical evaluation of the resin, specifically compressive strength, was conducted after the anti-icing test, as shown in Figure 6. The results indicate a noticeable reduction in compressive strength with the incremental addition of the anti-icing material (Mix 7) into Sample 5. The plain sample (unmodified) exhibited the highest compressive strength of approximately 30 MPa, confirming its robust mechanical structure in the absence of anti-icing additives [18,32]. However, as the anti-icing material was added in increasing amounts, a gradual decline in compressive strength was observed. At low concentrations (Samples 1–4), the compressive strength remained relatively stable, showing values between 28 and 30 MPa, suggesting that the resin’s structural integrity was initially well-preserved. This stability highlights the compatibility of Mix 7 with the resin matrix at lower additive ratios. However, as the concentration of the anti-icing material increased further (Samples 5–10), the compressive strength decreased significantly, with the lowest value recorded around 16 MPa. This reduction can be attributed to the higher content of magnesium chloride hexahydrate and other salts, which may disrupt the polymer network, reduce cohesion, and introduce localized weaknesses in the material structure [33]. Despite the decline in compressive strength at higher additive concentrations, it is important to note that the anti-icing performance, particularly the reduction in melting time, was enhanced (as seen in the corresponding trend for melting time in the figure). This trade-off suggests that while higher amounts of anti-icing material improve melting efficiency, they compromise the mechanical stability of the resin.

## 4. Conclusions

This study demonstrates the potential of methyl methacrylate (MMA)-based resin composites as an innovative and sustainable solution for enhancing road safety during winter conditions. The integration of anti-icing additives, such as magnesium chloride hexahydrate, and high-friction aggregates into the MMA resin matrix has proven effective in addressing the dual challenges of ice adhesion and skid resistance. The optimized formulation exhibited significant anti-icing performance, achieving a freezing point reduction to −12.9 °C and improved melting behavior while maintaining compressive strength above 16 MPa at higher additive concentrations. Skid resistance was enhanced by incorporating high-friction aggregates, ensuring reliable traction under icy and wet conditions. Despite the trade-off between mechanical strength and anti-icing efficiency at higher additive levels, the results underscore the feasibility of achieving a balanced formulation that meets both performance and durability requirements. The use of MMA composites offers several advantages over traditional deicing methods, including reduced environmental impact, improved durability, and ease of application. The economic viability of the proposed MMA resin-based composite lies in its durability and reduced maintenance requirements. Traditional road surfaces often require frequent reapplication of deicing chemicals, leading to recurring costs and environmental damage. By contrast, the enhanced anti-icing and wear-resistant properties of the MMA composite reduce the need for maintenance and chemical treatments. Although the initial material cost may be higher, the extended service life and reduced environmental impact present a cost-effective solution over the pavement lifecycle. Future work should focus on large-scale field trials and the development of standardized testing protocols to validate the long-term performance of these materials under real-world conditions. These efforts will support the broader adoption of MMA-based systems for winter road safety applications. The integration of recycled materials, such as reclaimed asphalt pavement (RAP) or waste polymers, into the MMA resin-based composite, could enhance its sustainability while reducing material costs. Preliminary investigations suggest that the inclusion of recycled aggregates or fillers may not compromise mechanical or anti-icing properties.

## 5. Future Studies

Future work should focus on large-scale field trials and the development of standardized testing protocols to validate the long-term performance of these materials under real-world conditions. The acoustic performance of the MMA resin-based composite, including its potential impact on rolling noise levels, remains an area for future investigation. Changes in texture, surface roughness, and material composition could influence both sound absorption and reflectivity. Evaluating these properties would be particularly important for applications in urban environments where noise pollution is a concern. Future work will focus on optimizing the formulation of the MMA resin-based composite to enhance its performance. The incorporation of nanomaterials, such as silica nanoparticles or carbon nanotubes, could improve wear resistance, mechanical strength, and skid resistance. Additionally, field trials under varying climatic conditions will be conducted to validate laboratory results. These efforts aim to establish a comprehensive understanding of the material’s long-term behavior and ensure its practicality for widespread application.

## Figures and Tables

**Table 1:** MMA-based resin Mix design.

	All Units in (g)						
Id	Methyl Methacrylate	Butyl Acrylate	2-Hydroxyethyl Methacrylate	2-Hydroxy-4-(octyloxy) Phenyl Methanone	Paraffin Wax	1-Dodecyl Mercaptan	Dimethyl-p-Toluidine
S1	31	-	-	0.8	2	4	0.2
S2	31	34	-	0.8	2	4	0.2
S3	31	**-**	34	0.8	2	4	0.2
S4	31	17	17	0.8	2	4	0.2
S5	31	16	18	0.8	2	4	0.2

**Table 2:** Heat of solution and freezing point results.

Content	Heat of Solution (KJ/g)	Freezing Point (°C)
Mix 1	0.445	−11.9
Mix 2	0.355	−12
Mix 3	0.165	−10.7
Mix 4	0.069	−10.8
Mix 5	0.258	−11.4
Mix 6	0.315	−12.1
Mix 7	0.429	−12.9

**Table 3:** Anti-icing materials mix ratio.

Content	Ratios (%)			
	Magnesium Chloride Hexahydrate	Potassium Acetate	Potassium Carbonate	Polyethylene Glycol
Mix 1	50	-	50	-
Mix 2	50	-	-	50
Mix 3	-	50	50	-
Mix 4	-	50	-	25
Mix 5	20	30	25	25
Mix 6	30	20	25	25
Mix 7	40	10	25	25

## Data Availability

The data presented in this study are available on request from the corresponding author. The data are not publicly available due to privacy.
